# Remain or return? The effect of social network and engagement on settlement intentions among high skilled migrants in Northeast China

**DOI:** 10.1371/journal.pone.0320013

**Published:** 2025-03-19

**Authors:** Wenbin Wang, Xiaohan Li

**Affiliations:** School of Philosophy and Sociology, Jilin University, Changchun City, Jilin Province, China; Zhejiang University, CHINA

## Abstract

China is transitioning from an immigrant-exporting country to an immigrant-importing country. To attract and retain global, it is necessary to analyze the factors influencing the settlement intentions of high skilled migrants to formulate policy support and tailor the management of transnational communities to their characteristics. This study used the Changchun sample from the Survey on Foreign Residents in China (SFRC 2017–2019) to examine the influence of social networks and social engagement on settlement intentions. It also considered differences in the classification of social networks and engagement strength, as well as classification by country of origin. This study identified that social networks and social engagement in the destination country play an essential role in the willingness of high skilled migrants to stay in Changchun. Additionally, international enclaves negatively affected their willingness to stay. Furthermore, the relative strength of migrants’ social networks and social engagement in the destination country exerted varying effects on the intensity of their settlement intentions. That is, using high skilled migrants with strong Chinese social networks and social engagement as the reference group, relying solely on a strong social network in China while lacking social engagement significantly reduced their willingness to settle. A weak social networks and low social engagement also significantly decreased their willingness to settle. Moreover, settlement intentions of the high skilled migrants differed significantly by country of origin. If we selected the same reference term, the willingness of expatriates from developed countries to stay decreased significantly. In the sections concerning comparison and robustness checks, national data were employed (SFRC 2019). Highly skilled expatriates in Guangzhou, Hangzhou, and Yiwu demonstrated the synergistic interaction between ethnic communities and work units (*danwei)*, whereas those in Changchun exhibited a certain uniqueness, embedding themselves in Chinese society through their own human capital and *danwei* affiliations.

## Introduction

As globalization continues and the free flow of human capital deepens worldwide, China is gradually shifting from a migrant-sending country to a migrant-importing country. There are two main categories of foreign laborers coming to China: small traders, temporary workers, and asylum seekers, as in the Chungking Mansions in Hong Kong, Sanyuanli, and Xiaobei Road in Guangzhou, known as representatives of low-end globalization [1]; and high skilled migrants represented by executives, professional technicians, and academics from multinational companies, seen as an essential driving force for national and regional competitiveness [2]. Some scholars argue that foreign migration to China is due to economic prospects; therefore, the migration processes are not necessarily sustainable. First, new changes have emerged in transnational mobility worldwide, including increased diversification, increased uncertainty, and the transition from long-term to temporary migration [3]. Additionally, qualitative studies by Chinese scholars on foreigners coming to China have shown significant commonalities in their adaptation strategies: superficial integration and deep separation [4]; transient households [5]; and the tendency to construct a home country’s social networks and form relatively closed small societies, form aggregated social spaces, and rarely participate in local activities [6]. Following the above, this study posed a new question: What is the future mobility strategy of expatriates who have specific behaviors in social networks and social participation, especially for highly skilled individuals? In other words, what are the mechanisms influencing the “stay” and “flow” of managers and professionals who are backed by world-class platforms and have an excellent educational background?

This question is posed as an advancement of the current research on the social integration status of foreigners in China, including an expansion of research on the integration and transnationalism of more diverse immigrant groups worldwide [7], and an empirical inquiry into the attraction and residence issues of migrants that the Chinese government has been highly concerned with. As human capital is critical in global innovation activities [8], getting international high skilled migrants to play a proper role depends not only on who will migrate, but also on who will stay [9], and this needs to be reflected through expatriates’ willingness to stay. Although intention to stay does not represent the final migration result, it can be regarded as a necessary criterion for action. For instance, foreign immigrants who want to stay make relevant decisions that provide clues to predict their mobile direction [10]. Research from Switzerland [11] shows that settlement intention predicts the next direction of mobility for migrants. Moreover, and even more importantly, settlement intention directly affects migration behavior and is increasingly synergistic with mobility behavior. Second, settlement intention has great significance; it represents perceptions and attitudes toward past migration experiences and social networks.

In previous studies, it has been rare to study China from the perspective of a migrant-importing country, not to mention the Northeast region, the most modern and wealthiest region in the 20th century. Amidst the planned economy, urban development is usually closely linked to a large state-owned enterprise (SOE), and the overlap between SOEs and urban communities creates the characteristic of “one city, one enterprise.” Although SOEs have participated in separating government and enterprises and in market-oriented reforms, they retain the national administrative hierarchy and has a strong *danwei* (work unit) character. First Automotive Work (FAW) group and Jilin University hold the top resource platforms and have the strength to invite high skilled migrants, providing excellent support to foreign experts coming to Changchun. In addition, Changchun, also known as China’s Detroit, has a well-developed automobile production industry. However, it has run into trouble in recent years, facing transition problems and population loss [12].

Based on the summary above, the definition of high skilled migrants in this paper is based on the “Classification Criteria for Foreigners Coming to Work in China,” issued by the State Administration of Foreign Experts Affairs of China, specifically focusing on top-tier talents in Category A and foreign professionals in Category B. This study used data from the Survey on Foreign Residents in Changchun (SFRC 2017–2019) and national data to examine how social networks and social engagement influence high-skilled immigrants’ willingness to stay through the lens of transnationalism and perspectives of immigrants’ social networks. The core questions were as follows: What are the mechanisms through which social networks and social engagement impact the inclination of highly skilled migrants to remain? This central query can be further subdivided into the following questions: Which characteristics of the population are more likely to influence a residential decision? How do groups differ by country of origin? Additionally, what distinctions exist among high skilled migrants residing in various Chinese cities?

### Theoretical background and previous findings

Immigrant social network theory is an essential to understand the spatial aggregation of immigrants. Proposed by Professor Douglas S. Massey and his collaborators, the theory refers to a series of social relations that transnational immigrants form owing to family ties and geography before and after migration, at the places of outflow and inflow. Individuals can leverage these ties to access essential resources, such as information, emotional support, and material assistance, which can significantly reduce the costs and risks associated with migration [13].

Schiller et al. questioned the role of the nation-state in regulating the activities in which migrants engage and the identities they create [14]. The study of transnationalism recognizes two phases. Scholarly research initially focused on a single geographical location, such as the country of immigration, to examine the discourses on the concept of “home” and a sense of “belonging” associated with the home country [15]. At that time, the direction of migration primarily flowed from the developing to the developed countries [16]; thus, relevant studies mainly focused on developed countries. Around 2014, scholars began approaching transnationalism by jointly studying home and residence countries [17]. The core of transnationalism comprises the everyday lives, activities, and social relations of transnational migrants [14].

This argument suggests that migrants, through their social relations and political actions across national borders, dynamically shape culture in terms of identity and break with the traditional analytical framework, which assumes that migrants belonging to a single society. Mazzucato has points out that migrants often belong to ‘two societies at the same time’, maintaining close ties with their countries of origin while gradually integrating into their country of residence [18]. This practice of crossing national boundaries provides a fresh perspective for analyzing migrants’ social networks and social participation patterns.

Social networks provide individuals with access to social contacts and support resources. Social participation requires strong initiative and external opportunities, requisites that highlight its potential to complement or compensate for a weaker social network in certain contexts. As Glick Schiller et al. pointed out based on their study on transnationalism, transnational migrants in their new place of residence engage in complex cross-border activities that create, shape, and potentially transform their identities [14]. These activities reflect migrants’ geographical mobility and underscore the critical role of social engagement, which provides migrants with a platform to integrate into local society through participation in community activities, volunteering, and cultural exchanges. In turn, these activities reshape their sense of belonging and identity. Therefore, research on social engagement elucidates migrants’ adaptation processes in their new residence and demonstrates how they balance local integration and connections to their home country in a transnational context.

Immigration has multiple social bases, among which the emergence of immigrant networks is probably the most paramount [16]. Previous studies have examined the effects of social networks and engagement in immigrants’ place of residences. A social network is a system of associations that individuals establish through social relationships involving relatives, friends, colleagues and neighbors [13]. The strength and extent of social networks directly affect an individual’s ability to access resources, information, and support. Social engagement refers to the degree of an individual’s involvement in community life. It encompasses activities such as volunteering, cultural events, political participation, and engaging with the community in other meaningful ways [19]. Social networks provide individuals with access to social contacts and support resources, while social participation can complement or compensate for a weaker social network in certain contexts provided that the individual shows strong initiative and has access to and capitalizes on external opportunities.

Korinek et al. [9] asserted that a social network in the place of immigration promotes immigrants’ transition from temporary to permanent residences. Min [20] described how Chinatown helps new immigrants build new social networks and provides other social support to allow those who lack a formal education and language skills to access residency and upward mobility. Wanner [11] investigated immigrants’ willingness to stay in Shanghai in terms of their migration experience and the economic, sociocultural, and psychological integration of foreign immigrants. A country’s social network composition is believed to affect expatriates’ willingness to stay. The degrees of adaptation to and satisfaction with the city of residence are also important indicators. Guo et al. [21] explored the social integration and residential intentions of foreign immigrants in Xi’an and found that their social networks of foreigners and social interactions in the local area can effectively contribute to the settlement intentions of foreigners in China. This finding aligns with our research focus on the influence of social engagement and social networks on settlement outcomes.

Reyes [22] believed that social interactions in a destination country could positively influence residence, a link that highlights the role of local social engagement in fostering a sense of belonging. Furthermore, a study conducted in the Moabit West neighborhood of Berlin with a focus on Turkish and Arabic immigrants, indicated that social participation, particularly in community activities, plays a crucial role in enhancing communication between immigrant and native groups, ultimately fostering better immigrant integration and increasing their likelihood of establishing long-term residence [19].

Research on immigrant social participation differentiates between formal and informal involvement. Formal participation typically involves joining organizations or collaborating with institutions, whereas informal participation entails self-organization or self-directed engagement. A study examining Polish immigrants in nine European countries found that they often prefer informal modes of participation [23]. Carling and Pettersen [10] argued that the relative strengths of immigrant integration and transnationalism are determinants of naturalized immigrant intentions, and are neither correlated predictably nor independent of each other. Both these determinants co-participate in shaping migrants’ intentions. Specifically, immigrants with solid transnationalism but weak integration into their country of residence are more likely to return to their origin countries. Conversely, strong social integration and the concomitant weakening of transnational ties in an immigrant’s destination country would reduce the likelihood of return to their origin countries. However, some scholars have challenged this claim, arguing that the return of migrants may also be an outflow after successful integration in the inflow country [24].

Understanding these complex processes requires a framework that connects global economic processes with migrants’ social relations and cultural identities. Glick Schiller et al. emphasized that migrants’ social relations are fluid and dynamic, as well as deeply influenced by cultural patterns. This dynamism suggests that migrants’ social networks can be fluid and reconfigured in transnational contexts, while social participation is an important channel through which migrants can reestablish a sense of belonging to their country of residence. Therefore, social networks and social participation are two key variables in migration studies, and should not be analyzed in isolation, but rather should be linked in the dynamic context of globalization, for an increasingly comprehensively understanding on how migrants balance local integration with their country-of-origin ties.

In summary, the main field sites of Western studies are concentrated in South-North flows, which contrasts with the unique characteristics of expatriates in China. However, research on expatriates in China are limited, despite the country having emerged as a top destination for highly skilled migrants in recent decades. In the context of Chinese scholars, there are also the following characteristics: first, qualitative studies dominate the existing literature, whereas quantitative studies are few. Second, among these limited quantitative studies, some indicators were replicated from domestic mobility groups, such as whether they are self-employed or employed, or whether they own or rent housing. These indicators do not capture the specific experiences of transnational migrants. Furthermore, the experience of internal mobility cannot be generalized to overseas migrants because the challenges and opportunities faced by expatriates in a foreign country vary distinctly different from those encountered by domestic migrants. Third, large-scale social surveys and databases involving foreigners are limited to megacities, such as Beijing, Shanghai, Guangzhou., and Hangzhou, leaving a significant gap in understanding the settlement patterns of expatriates in mid-sized cities, such as Changchun. As mentioned previously, the regional characteristics of Changchun are significantly different and have been shaped by its industrial base, government-enterprise-university collaborations, and a relatively concentrated pool of high skilled migrants. The government and large multinational companies such as the FAW Group and Jilin University have formed a stable government-enterprise-university triple helix structure. This structure attracts considerable many high skilled migrants to transnational immigration, particularly in the automotive and engineering sectors, which offer for transnational employment opportunities. Hence, high skilled migrants in Changchun exhibit significant unitary, concentrated, and regional characteristics [25].

These complementary perspectives help explain how first-generation transnational immigrants manage their settlement processes in China. Given that transnational immigrants working in China are first-generation immigrants, among those born abroad, their home country-based social network and activity participation experiences will likely impact their settlement intentions [26]. Therefore, this study built a research framework from the perspectives of immigrant social network theory and transnationalism. Integrating transnationalism with social integration theories provides a comprehensive lens for understanding the settlement intentions of first-generation immigrants. Transnationalism emphasizes immigrants’ dual embeddedness in their origin country and resident country, highlighting the role of transnational social networks and activities in shaping their experiences. Moreover, social integration focuses on the migrants’ engagement with and adaptation to the host country’s social systems, such as their participation in local networks, community activities, and economic structures. These two frameworks are not contradictory but complementary. While transnational ties may influence immigrants’ initial decisions to migrate and their continued connection with their home country, social integration determines their degree of embeddedness and long-term intentions to settle in the host country. Together, these allow us to explore how immigrants’ settlement intentions are shaped by the interplay between maintaining transnational networks and engaging with local structures.

This study combined social networks and the degree of social engagement to propose four typologies of migrant behavioral patterns: strong social networks with high social engagement (SNSE), strong social networks with low social engagement (SNWE), weak social networks with high social engagement (WNSE), and weak social networks with low social engagement (WNWE). This resultant framework encompasses a spectrum ranging from highly integrated to relatively isolated behavioral patterns.

The introduction of this framework marks a shift from single-variable studies to multidimensional integrated analysis, laying a new theoretical foundation and providing a fresh analytical pathway for future research. By constructing such an integrated analytical framework, this study enriches the theoretical dimensions of transnationalism through the organic combination of the strength of migrants’ social networks with their level of social engagement. It provides a novel approach to exploring how migrants balance local integration with connections to their home countries in a transnational context. Furthermore, this framework reveals the dynamic mechanisms underlying diverse migrant behavioral patterns, thereby offering a systematic theoretical tool for understanding the social adaptation processes of different migrant groups. Additionally, this framework has significant practical implications for designing migrant support policies. By identifying the social needs and behavioral characteristics of different types of migrants, policymakers can formulate more targeted support systems to promote integration and long-term development among migrant populations.

The social, economic, and cultural aspects of immigration do not always align perfectly within a given geographical area. Instead, immigrants actively participate in the socio-economic activities of both their home and host countries, resulting in the movement of people, goods, and capital between nation-states. This study proposes research hypotheses based on the social network theory of immigrants and the transnationalism perspective. It explores the factors influencing the willingness of high skilled migrants to stay in China in three dimensions: origin country and China, social network strength and level of social engagement, and type of origin country.

### Hypothesis of the facilitative effect of social network variability on settlement intentions

Globalization, in the context of transnational dispersal, has accelerated the phenomenon of ethnic diaspora on a larger scale worldwide. People from different countries reorganize groups, reconfigure history, and reset the community’s imagination. Migrant networks link the place of origin to the place of inflow; the social network of immigrants has generally been divided into one of immigrants whose social contacts are mainly foreigners and one of immigrants whose social contacts are mainly Chinese in the existing literature. Moreover, the origin-based immigrant social network dominated by foreigners can be divided into an international-oriented social network and a home-country-oriented social network.

First, the social networks of immigrants with Chinese social networks have several characteristics: a closed social circle, shallow integration, and deep separation; some groups form aggregated spaces and maintain a certain distance from mainstream Chinese society. Therefore, it is assumed that immigrants expect to return to their country of origin or plan to move to other countries in the next step. Under such conditions, their social networks and participation may be limited or highly selective, and they may not be willing to learn about or participate in local social activities. There is also little incentive to learn the destination country’s language, and they are more inclined to make friends in their own country or have international friends.

Second, there is a more specific category of departure-attributed immigrants who are ethnic-attributed through blood, hometown, and affection ties, which reflect a strong preference for gregariousness and play an essential role in helping immigrants adapt to the culture shock, providing information and jobs. Strong ties help immigrants expand their social networks and adapt to life in Guangzhou. Additionally, the social networks of African businesspeople in Guangzhou are reconstructed through social participation. For example, Xiaobei Road in Guangzhou, Gubei in Shanghai, Wangjing in Beijing and other transnational immigrant settlements have well-developed ethnic networks and economies. They are self-sufficient in terms of commerce, healthcare, and education.

Finally, interethnic contact is often considered an essential indicator of cross-cultural social integration [27]. A previous study showed that ChinaNet can significantly promote the social integration of international immigrants [25] and that Chinese social networks can also significantly increase international immigrants’ willingness to stay. In the 19th century, the United States had already started research on “cross-cultural adaptation,” mainly focusing on the adaptation after immigrants enter a new cultural environment. Interactions with the mainstream society are important indicators. High skilled foreigners who intend to stay are motivated to invest in a localized Chinese social network and actively integrate into China through several social activities. Those who find interethnic contact in Chinese society attractive to continue living in Changchun may be more inclined to stay longer. However, in this case, it may also have the opposite effect of shattering the original imagination during actual interethnic contact. Thus, interethnic contact may prompt them to refuse long-term residence.

In brief, the composition of immigrants’ social networks shows some variability in enhancing the continuity of high-skilled immigrants. With reference predominantly to international expatriates, ethnicity, and Chinese social networks, this study formulated the following hypotheses regarding an imaginary international community, ethnic social networks, and mainstream cultural adaptation.

H1a: The more substantial the international social network of Changchun’s high skilled migrants, the stronger their willingness to stay in China.

H1b: The more substantial the ethnicity of Changchun’s high skilled migrants’ social network, the stronger their willingness to stay in China.

H1c: The more substantial the Chinese attributes of Changchun’s high skilled migrants’ social network, the stronger their willingness to stay in China.

H1d: The higher the concentration of high skilled migrants residing in international enclaves, the less likely they are to stay in China.

H1e: The higher the concentration of high skilled migrants residing in ethnic enclaves, the less likely they are to stay in China.

### Hypothesis of the promotion effect of social engagement on settlement intentions

The main constituent groups of overseas high skilled migrants in Changchun are managers, technical business directors, and university researchers who hold legal residency status, possess substantial human capital, and enjoy a high socioeconomic status. Most of them in Changchun serve the city’s key industries and its most resource-rich platforms, such as the FAW Group and its subsidiaries, universities, and research institutes, including Jilin University, Northeast Normal University, and the Institute of Optics and Mechanics, which can be called *danwei* (work units). Consequently, these migrants secure advantageous positions and access to derived resources. During the planned economy era, individuals within *danwei* received extensive corporate welfare and social support, including housing, children’s education, medical care, and access to community facilities such as canteens, bathrooms, and cinemas [28]. While the *danwei* system has diminished since the reform of state-owned enterprises, it continues to offer formal social support for high skilled migrants through professional networking, financial assistance, housing, and education for their children.

It has been shown that people with a high socioeconomic status are typically more expressive, independent, and socially engaged [29]. Expatriates in Changchun are members of a transnational community that exists in both local and global contexts [28]. Cumulative causal models based on social networks also indicate that migrant behavior is influenced by outgoing and incoming countries, often using the number of connections between outgoing and incoming countries as a measure.

Hence, this study examined the types and degrees of social engagement among high skilled migrants in their places of departure and in China. Engagement was categorized into formal and informal types. In their place of departure, high skilled expatriates may formally participate in high-level political activities, such as voting and joining political parties, or participating in associations, including anti-government organizations. Nevertheless, due to the limitations like nationality and other restrictions, it is not possible to make a one-to-one correspondence of the levels of social participation in places where high skilled migrants move. However, the distinctions can be made between formal and informal participation. Therefore, this study explored the participation of Changchun high skilled migrants in the chambers of commerce (1 The “Interim Provisions on the Administration of Foreign Chambers of Commerce” are as follows: 1. The name of a foreign chamber of commerce should be titled with the name of its own country plus the word “China”; 2. To establish a foreign chamber of commerce, a written request should be submitted to the Ministry of Civil Affairs of the People’s Republic of China (hereinafter referred to as the registration authority). Apply and register in accordance with the law. The registration management organ shall make a decision on whether to approve the registration within 60 days from the date of receiving all the documents specified in Article 8 of these Regulations, and issue a registration certificate if the registration is approved; and explain the reasons in writing if the registration is not approved. A foreign chamber of commerce is established upon approval and registration and the issuance of a registration certificate; 3. The foreign chamber of commerce shall accept the supervision of relevant Chinese authorities.) and volunteer activities. Since foreigners’ chambers of commerce and hometown associations in China must be registered, invited, or supervised by formal organizations above a certain level under Chinese law, these activities involve formal participation. Conversely, volunteer gatherings and joining associations have a lower threshold of entry are classified as informal participation.

Research has shown that social engagement in the home country is consistently and positively correlated with all types of return intentions [30]. Accordingly, a highly skilled expatriate in Changchun who sees their *danwei* as a “project-based” platform and cares more about their hometown’s social experience, political life, and social life may prefer to move back to their home country. They may then be more inclined to return to their home country, which is negatively correlated with their stay in Changchun, China. However, there may be mechanisms in the opposite direction. If they weigh the information, resources, and social support they receive by staying in Changchun and believe that their aspirations upon return are somewhat different from or insufficient for their actual situation in their home country, then a willingness to stay may also emerge.

To investigate the influence of social engagement in the place of departure and immigration on willingness to stay, the following hypothesis was proposed:

H2: The stronger the social engagement of high-skilled immigrants in their place of migration, the stronger their willingness to stay in China.

### Social engagement–social network matrix hypothesis

Given the increased and increasingly diverse patterns of international migration flows, scholars have explored ethnically diverse research hypotheses and influences on the “stay” and “flow” of transnational migrants [31,18,32]. There are competing and contradictory relationships among these reasons for return [30], which cannot provide reasonable explanations or predictions for China’s transnational migration.

Based on a systematic discussion of expatriates in Guangzhou, Beijing, Shanghai, and Yiwu by Chinese scholars, we identified four ideal types of social networks and social engagement. The first category comprises expatriates with strong Chinese network attributes and strong local engagement (SNSE). These individuals possess abundant social capital, which enables them to transcend geographical boundaries and become barrier-free migrants [8]. In the framework of transnationalism, they are simultaneously rooted in their place of origin, place of emigration, and sometimes even in a third country [18]. In China, most people live alongside local residents, and many take on roles as expatriate volunteers, social workers, and mediators within the community, actively exhibiting strong economic integration and social embeddedness.

The second category comprises expatriates with weak Chinese social networks and weak local engagement (WNWE). To some extent, they have rich international and ethnic network resources and live far from mainstream society under the dual effects of the active aggregation of cultural and ethnic economies and the mechanism of social exclusion. Unlike the former, who have vital Chinese social networks and solid social participation, they depend more on ethnic networks. They are likely to refrain from remigrating if they can form a self-sufficient comfort zone in their place of residence.

The third category includes expatriates with weak Chinese social networks and strong local participation in the place of immigration (WNSE). They care more about the personality characteristics of their social contacts than just about their common nationality or similar migration experiences. Some of them have established relatively large-scale enterprises that have become the well-known “China Hands” and a bridge between the Chinese and their home countries; it is believed that their willingness to settle in China is relatively strong.

The fourth category includes expatriates with a strong Chinese social network and weak local participation in the place of immigration (SNWE). They generally use the Chinese as an intermediary to help them adapt to the local mainstream culture, handle various affairs, and maintain a certain social distance from the local society. This group is likely to be attracted to China’s economic form and will also tend to seek better platforms and economic gains.

Therefore, we considered the relative strength of social networks and engagement as cross-dimensions that can be represented by a matrix containing four categories. These include expatriates with SNSE, SNWE, WNSE, and WNWE. Taking expatriates with SNSE as a reference, the following assumptions were made to predict the flow direction:

H3a: Immigrants with strong Chinese social networks and weak social participation characteristics (SNWE) have stronger settlement intentions.

H3b: Immigrants with weak Chinese social networks and strong social participation characteristics (WNSE) have stronger settlement intentions.

H3c: Immigrants with weak Chinese social networks and social participation characteristics (WNWE) have stronger settlement intentions.

In addition, the demographic composition of the migrant group, migration history, current and future economic development of the country of departure, cultural attributes, and social background may have influenced expatriates’ willingness to stay [33]. In their study of returning Chinese expatriates in Guangzhou, Liang and Huang [34] revealed different migration mechanisms between developing and developed countries and that returning Chinese expatriates from developing countries have a stronger intention to stay than those from developed countries. From this, it is assumed that there will be some variability between high skilled migrants with North–South mobility and high skilled migrants with South–South mobility, from which the hypothesis of the country of origin of migrants can be further assumed:

H4: Country-of-origin differences exist in the willingness of high skilled migrants to stay in China, and highly skilled migrants whose countries of origin are developed have a lower willingness to stay in China.

### Data sources, variables, and statistical description

#### Data.

The Survey on Foreign Residents in China (SFRC), initiated in 2017, is a comprehensive and continuous large-scale cross-sectional survey conducted by the Sun Yat-sen University Social Science Survey Center. It is conducted in seven cities: Guangzhou, Changchun, Hangzhou, Yiwu, Xi’an, Lanzhou, and Xuzhou. The SFRC targets all foreigners aged 18 years and above who enter the Exit-Entry Administration Bureau during a specific period. The survey is a large-scale questionnaire on the basic information of foreigners before coming to China; their migration process; life, work, residence, and social network after coming to China; economic dependence; participation in social activities; cross-cultural adaptation; and willingness to stay. During the investigation, we underscored the voluntary nature of participation by explicitly informing the respondents that their engagement was entirely at their discretion.

According to the Law of the People’s Republic of China on the Exit and Entry Administration, foreigners with visas stating that they must apply for a residence permit after entry shall apply for one at the Ministry of Public Security of the People’s Republic of China within 30 days from the date of entry. Thus, sampling collected at the exit and entry administration bureau better represents foreigners in China because it is mandatory for foreigners to stop upon arrival in China or before the expiration of their visas. In addition, we provided questionnaires in 13 languages for respondents to choose their preferred language and conducted a long-term survey over two months to achieve more robust coverage and allow participants the flexibility to respond at their convenience.

The data in this paper came from the “Survey on Foreigners Living and Working in Changchun” conducted by the Department of Sociology of Jilin University at Changchun Exit-Entry Administration Center from 2017 to 2019. According to the “Classification Criteria for Foreigners Coming to Work in China,” the emphasis lies on top-tier talents in Category A and foreign professionals in Category B. Based on observations from Changchun, where foreign high-tech immigrants are predominantly employed in *danwei* (work unit) such as the FAW Group and its subsidiaries, universities, research institutes, and educational institutions, high skilled migrants encompassed managerial, scientific research, and professional roles; consequently, 187 individuals were included in the sample.

To understand the uniqueness of Changchun, the data from Guangzhou, Yiwu, and Hangzhou were compared. In total, 777 samples were included in the regression analysis.

#### Dependent variable.

The dependent variables in this paper were settlement intentions, including “immigration intention” and “desired length of stay.”

First, immigration intentions were measured by “Have you ever considered submitting an application to immigrate to China this time you come?” Respondents could choose from the following options: (1) never thought about it, (2) I considered it before I first came to China, (3) I considered it during my stay in China, and (4) other. Since the second and third categories of respondents had considered applying to the Chinese government to immigrate, the responses were combined into a single category of “‘intention to stay.”

Second, “desired length of stay” was determined by “How long do you wish to stay in China this time?” Respondents could choose from the following options: (1) leave as soon as possible, (2) stay as long as possible, (3) permanent residence, or (4) others. Since both “stay as long as possible” and “permanent residence” involved individuals considering long-term or even permanent residence, these answers were combined into one category, “willing to stay.”

Finally, this paper regarded “intention to stay” and “willing to stay” as expatriates having the desire to stay, treated as 1. In contrast, if there was no willingness to stay, it was classified as 0. Thus, we constructed a dichotomous variable for settlement intention.

To avoid operational biases, we used the question, “How long do you intend to stay in China this time?” for re-measurement in the section of the comparative analysis and robustness check, with “leave as soon as possible” designated as 1, “stay for a longer period if possible” as 2, and “permanent residency” as 3, thus forming a quantitative measurement of settlement intention strength.

#### Independent variables.

For the independent variable of social networks, we first considered size and composition. The generated expatriates knew the number of Chinese people, their countries’ people, and people from other countries (not including those from their own country). The options “(1) 1–5, (2) 6–10, (3) 11–20, (4) 21–50, (5) 51–100, (6) 101–150, (7) 151–200, (8) 201–250, (9) 251–300, (10) 301–350, (11) 351–400, (12) 401–450, (13) 451–500, (14) >500, and (15) 0” were normalized into a range from 1 to 5 after squaring the corresponding numbers. Values exceeding 2.5 times the standard deviation were compressed to a size of 2.5 times the standard deviation, resulting in the final measurement of the network scale on Chinese Social Networks (CSN), Ethnic Social Networks (ESN), and Global Social Networks (GSN).

Then, the degree of immigrant clustering is measured by asking: “How are international immigrant clusters distributed in the area where you reside?” and “How is the distribution of people from your home country in the area where you reside?”

The operational approach to the other core independent variable, social engagement, was measured using the following questions: “Have you participated in any business associations/ethnic-related gatherings in China?” and “Have you participated in any volunteer gatherings in China?”, where ‘Yes” was coded as 1 and “No” as 0. The responses were then summed to obtain the variable for participation in China. Similarly, the questions “Have you participated in elections in your home country?” and “Have you participated in any associations in your home country?” were used, with “Yes” coded as 1 and “No” as 0, to obtain the variable for participation in the home country.

#### Control variables.

In the statistical model, gender, age, education level, survey year, and residence time were introduced as control variables; the details were handled as follows:

Gender: Male was assigned as 1, and female was assigned as 0.

Age: The actual age was calculated in 2023 based on the date of birth.

Education level: This was measured by the highest level of education.

Year: Value was assigned according to the survey year.

*Danwei* support: Based on the question, “Who was responsible for recepting you when you first arrived in China?”, unit reception was defined as one, and no unit reception was defined as zero.

### Method of analysis

We used R software for the logistic regression analysis to examine the settlement intentions of high skilled expatriates in Changchun. Logistic regression is suitable for binary outcome variables, making it appropriate for our study, in which settlement intention is dichotomous (stay/leave). Prior to conducting logistic regression, we performed data preprocessing steps, including handling missing values, outlier detection, and normalization, to ensure data quality and comparability. The selected occupations included managers, professionals in specialized fields, and technicians. We constructed a logistic regression model using social networks and participation variables as predictors. Exponentiated coefficients (odds ratios) were used to interpret the impact of predictors on the odds of having the intention to stay. We accounted for the potential impact of survey year on our findings by controlling for survey year in the regression analyses. We are aware of the importance of addressing temporal variations, and took rigorous measures to control these effects.

#### Descriptive statistics.

Table 1 shows that more than 72% of Changchun’s high skilled expatriates were male, with an average age of 39 years, which is the golden period of career development. Moreover, a vast majority had obtained university degrees or above. Regarding the countries of origin, 66.1% came from developing countries and 33.9% from developed countries.

**Table 1:** Descriptive Statistics of Related Variables, *N*=187, China, 2017–2019.

Variable		Percentage (%)	
Settlement Intention			
Yes		59.1	
No		40.9	
Sex			
Male		72.0	
Female		28.0	
Education			
Below College/University		7.5	
College/University or above		92.5	
Social engagement			
China Engagement Attribute (CEA)		7	
International Engagement Attribute (IEA)		93	
Support network			
*Danwei* support		27.3	
*Non-Danwei* support		72.7	
Survey Year			
2017		34.8	
2018		44.4	
2019		20.9	
Country			
developed		125	
developing		62	
Variable	**Maximum**	**Minimum**	**Standard Deviation**
Age	74	23	10.3
International enclave	0	5	1.37
Ethnic enclave	0	5	1.20
Chinese network	1	1.70	0.26
Ethnic network	1	1.06	0.02
Global network	1	1.28	1.11

The proportion of high skilled migrants with *danwei* reception upon their first arrival in China was 27.3. Among expatriates, 59.1% were willing to stay in Changchun. In comparison, the rest (40.9%) maintained the mentality of passers-by, who expected to return to their own country immediately after several years of reassignment. Regarding social network construction, the network sizes of the Chinese (1.35) and Chinese networks (1.61) were larger than the size of the home country networks (1.04). In terms of preference for social participation, 93% of high skilled migrants participated in social, economic, and cultural activities in their home country, which was far higher than their willingness to participate in the Chinese society (7%).

## Results

Through a contingency table analysis of various variables related to social engagement attributes and settlement intentions (Table 2), we found that the developmental level of the home country, Chinese language proficiency, hometown city size, income level of the home country, Chinese enterprise investments, educational experience in China, and family members’ work experience in China did not show significant correlations with settlement intentions. However, after arriving in China, the *danwei* support network exhibited a significant association with settlement intention.

**Table 2:** Contingency Table of Factors of Social Participation and Settlement Intentions, N=187, China, 2017–2019.

Settlement Intention				
**Variables**	Options	Yes	No	Chi-square test
**Development in Home Country**	Developed Countries	63	62	Chi2=0.011 p-value=0.917
	Developing Countries	30	32	
**Chinese Proficiency**	Fluent	67	61	Chi2=0.800 p-value=0.371
	Not fluent	26	33	
**Support Network**	Non-*Danwei* support	77	59	Chi2=8.473 p-value=0.004^***^
	*Danwei* support	16	35	
**Hometown**	Rural	9	11	Chi2=1.211 p-value=0.546
	Small City	33	39	
	Large City	51	44	
**Home Country**	Low Income	13	12	Chi2=0.678 p-value=0.878
	Low-Middle Income	29	29	
	Middle-High Income	16	13	
	High Income	35	40	
**Chinese Enterprises Investment**	None	6	13	Chi2=2.038 p-value=0.1534
	Yes	87	81	
**Education in China**	None	56	66	Chi2=1.643 p-value=0.199
	Yes	37	28	
**Family Members’ Work Experience in China**	None	73	67	Chi2=0.940 p-value=0.333
	Yes	20	27	

This study used logistic regression (Table 3) to test the impact of social networks and social engagement factors of different dimensions in terms of both origin and residence. Based on the control variables, Model 2 incorporated the relevant variables: the social network of high-skilled migrants in Changchun, including Chinese network attributes, home country network attributes, Chinese network attributes, international enclaves, and ethnic enclaves. The variable included in Model 3 was social participation, as predicted using SoftMax. Model 4 incorporated both social network and social participation variables.

**Table 3:** Logistic Regression Analysis of Factors Influencing the Willingness of High Skilled Migrants to Stay, N=187, Changchun, 2017–2019.

	Dependent Variable: Settlement Intention			
	Model 1	Model 2	Model 3	Model 4
**Gender**	-0.163	-0.250	-0.104	-0.219
	(0.351)	(0.377)	(0.359)	(0.385)
**Survey Year 2018**	-0.048	-0.084	-0.055	-0.101
	(0.346)	(0.369)	(0.352)	(0.374)
**Survey Year 2019**	-0.281	-0.512	-0.347	-0.589
	(0.434)	(0.488)	(0.446)	(0.499)
**Age**	-0.027^*^	-0.022	-0.028^*^	-0.023
	(0.016)	(0.017)	(0.016)	(0.017)
**Education**	-0.211	-0.290	-0.335	-0.381
	(0.596)	(0.647)	(0.601)	(0.646)
**Country Type**	0.087	-0.530	0.063	-0.545
	(0.335)	(0.401)	(0.343)	(0.410)
***Danwei* Support**	1.102^***^	1.461^***^	1.169^***^	1.485^***^
	(0.355)	(0.394)	(0.362)	(0.397)
**Chinese Network**		-1.460^*^		-1.545^**^
		(0.756)		(0.778)
**Ethnic Network**		24.381^**^		23.307^**^
		(10.698)		(10.771)
**Global Network**		4.215^**^		3.917^**^
		(1.662)		(1.693)
**International Enclave**		-0.414^**^		-0.418^**^
		(0.162)		(0.166)
**Ethnic Enclave**		-0.151		-0.148
		(0.158)		(0.161)
**China Engagement**			1.979^**^	1.834^**^
			(0.802)	(0.841)
**Constant**	0.937	25.875^**^	0.937	-24.274^**^
	(0.810)	(10.729)	(0.817)	(10.808)
**Observations**	187	187	187	187
**Log Likelihood**	-123.026	-113.440	-118.869	-110.354
**Pseudo R-squared**	0.091	0.212	0.145	0.248

* p<0.1;

** p<0.05;

*** p<0.01.

### The influence of the social network on settlement intensions

Model 1 controlled for variables such as sex, age, education level, survey year, and *danwei* support. Specifically, gender difference and education in the settlement intentions of expatriates in Changchun were not statistically significant, but age (p<0.1) was statistically significant, with negative coefficients. The negative coefficients of age were consistent with life-cycle theory findings, which state that the older the age, the higher the migration cost. Additionally, the figure showed that the higher the educational level, the lower the willingness to stay. The *danwei* support was significantly positive (p<0.01).

Model 2 indicated that the size of social networks significantly influenced the settlement intentions of high skilled migrants. Specifically, ethnic and international social network sizes had a significant impact on the settlement intentions of high skilled migrants, whereas Chinese social networks had a significant negative impact.

Although the scale of their Chinese social network was larger than that of the ethnic network, the mainstream acculturation hypothesis was not established, indicating that highly skilled migrants in Changchun also have shallow integration characteristics and passing-by characteristics [5] that appear in other Chinese cities, possibly because of their living habits and cultural beliefs that are quite different from those of Chinese residents. This could also because they live in closed and homogeneous communities and, like global elites, form “closed groups that are detached from their local historical and cultural roots” [35] in terms of social status, economic status, culture, and space.

*Danwei* (work units) have established Foreign Affairs Offices or International Departments to facilitate better integration into mainstream Chinese society through housing, language assistance, visa services, and the organization of cultural and social events. Structured support and resources enhance international experts’ day-to-day living experiences and foster a sense of stability and security, which are vital factors in their decision to extend their stay in China.

However, this support cannot fulfil deeper emotional support and a sense of social belonging, especially in terms of interactions with Chinese people. First, the high skilled migrants in this study were first-generation migrants born abroad and working in China, who differ significantly from Chinese residents in terms of lifestyle habits and cultural beliefs. This disparity may lead to feelings of isolation or disconnection from the local culture. Second, their Chinese social networks are often superficial, characterized by “hi-bye” friendships or interactions limited to the workplace. Such emotionally and socially detached relationships rarely foster a sense of place attachment, which can diminish their willingness to stay in China over the long term despite the practical aspects of work and daily life benefits provided by their *danwei*.

High skilled migrants residing in enclaves with a higher concentration of international migrants tend to have a stronger desire to leave China. This result reflects the negative impact of ethnic enclaves, where the clustering of individuals with similar backgrounds can reinforce feelings of isolation from mainstream society. Instead of fostering a sense of belonging, these enclaves may inadvertently exacerbate the cultural divide, making it more challenging for international expatriates to integrate into local communities. However, the regression results for expatriates residing in ethnic enclaves comprising compatriots were not statistically significant. As transnational migrants in Changchun are primarily international students, relatively fewer individuals seek career opportunities and professional growth compared to those in other cities. Moreover, low-skilled transnational laborers are scarce, preventing the formation of a self-sufficient ecosystem that fully supports needs such as food, education, healthcare, and employment.

### The influence of social engagement on settlement intentions

Model 3, which controlled for essential attributes, examined the influence of social participation. Results showed that the level of social engagement among high-skilled professionals in China significantly influenced their willingness to remain. Research hypothesis 2 was verified; that is, high skilled migrants with more social involvement in China firmly intend to stay.

The study found that immigrants with higher economic and social levels also strongly desired social participation. Some cities have experience in collaborative co-management and governance, such as the Yangshuo Public Security Bureau’s Immigration Management Brigade, which has formed a foreign volunteer service team to hold regular symposiums to strengthen ties between foreigners from different countries, make use of foreign language advantages to help other foreigners with disputes, and assist in police mediation and other tasks. However, the current social participation rate of high skilled migrants in Changchun is only 7%, much lower than 93% in the country of origin, which proves considerable potential and urges more policies and activities to enhance and promote social participation.

### Settlement intention under the dual influences of social engagement and network

In the logistic regression, we measured the explanatory power of the separate variables on the intention to reside. In a study on immigrants’ willingness to return, Carling and Pettersen [10] found that the relative strength of ties to the country of residence and country of origin played a decisive role in their willingness to return. The high skilled migrants from Changchun have a wide range of origins. Although they have the same high skilled migrants assessment system, they have slightly different occupational and duty scopes. Hence, it is necessary to explore the differences between the possible combinations of strong and weak social engagement and networks in China.

The operationalization process was as follows: First, we used the machine learning function SoftMax to convert CSN and ESN into probability vectors, allowing for a comparison of the probabilities of high-tech professionals’ social networks in China and their home country. If the probability value for China was greater than that of the home country, then the professionals’ social network was assigned a value of 1, indicating “strong.” Otherwise, it was assigned a value of 0, indicating “weak.”

Then, the SoftMax machine learning function was employed to predict the China Engagement Attribute (CEA) and International Engagement Attribute (IEA) and to compare these probability values. If the probability of involvement in China was greater than that in the home country, it was set to 1, indicating stronger participation in China. Otherwise, it was set to zero, indicating a weaker involvement.

These strong and weak social participation and social networks were considered cross-cutting dimensions that a matrix can represent. This resulted in four ideal types of immigrants’ residential behavior (see Table 4):

**Table 4:** Bivariate Matrix of Social Participation and Chinese network Attributes.

Chinese Social Network (-)	Social Engagement in China (+)		Chinese Social Network (+)
Ⅱ**. WNSE** Weak Network and Strong Engagement	Ⅰ**. SNSE** Strong Network and Strong Engagement
Ⅲ**. WNWE** Weak Network and Weak Engagement	Ⅳ**. SNWE** Strong Network and Weak Engagement
**Social Engagement in China (-)**

(1) When both CSN and CEA were strong, they were recognized as SNSE. University teachers exemplify this. Their professional roles necessitate active engagement in a variety of institutional activities, such as academic teaching, mentorship programs, and research collaborations, cultivating close relationships with both students and colleagues, and facilitating the flow of knowledge and resources within the academic community. Beyond their institutional responsibility, their influence extends to a broader social sphere. They often participate in public lectures, industry partnerships, community outreach programs, and professional associations, thereby bridging the gap between academia and community. This dual embeddedness within academic institutions and larger societies makes them key players in promoting cross-sector collaboration and social cohesion.
(2) Individuals with a strong CSN but a weak CEA were classified as having SNWE. Expatriate executives in FAW represent this category. These executives typically reside in international neighborhoods provided by *danwei*; participate in activities; and establish strong ties with their colleagues, clients, and industry partners, allowing them to thrive within their professional roles and effectively navigate China’s business landscape. However, social interactions are often limited to work-related contexts, and they tend to remain relatively detached from broader local community activities or social engagement within the local cultural or social fabric.
(3) The WNSE category is exemplified by English teachers working in language institutions, especially those employed through intermediary agencies. These teachers are frequently assigned to multiple schools or training centers and work with students across a broad age range, from preschoolers to adults. The nature of their roles, structured by intermediary agencies, requires them to move frequently between schools, which limits their opportunities to build strong and stable social networks outside of work. Additionally, the need to adapt to various age groups demands considerable preparation and focus, often leaving teachers with limited time and energy to engage deeply with local communities.
(4) The WNWE category includes three main groups of expatriates. The first group consists of project-based experts. The second group within WNWE consists of experts who have retired from Germany or Japan and are rehired in China, often serving in advisory or consulting capacities. The third group includes accompanying spouses who move to China to support their partners, but continue their employment or affiliations with institutions abroad. The primary focus of these three groups is on fulfilling professional obligations or supporting a spouse rather than integrating into the local community, demonstrating minimal social ties and low levels of engagement.

This matrix is then used to predict and analyze the flow of immigrants after a specific social integration orientation and behavior.

Table 5 presents a regression analysis based on the relative strength of the four quadrants to explore the settlement intentions of different types of high skilled migrants in Changchun. A more refined analysis was then carried out by constructing models based on the classification of the countries of origin. Model 3 represents high skilled migrants from developed countries, while Model 4 represents high skilled migrants from developing countries who may face different socioeconomic backgrounds and challenges. The results are as follows (Table 5).

**Table 5:** Logistic Regression Analysis of Social Engagement and Chinese Network Attribute Matrix, *N*=187, Changchun, 2017-2019.

	Logit regression			
	*Settlement Intention*			
	Model 1	Model 2	Model 3	Model 4
**Gender**	-0.169	-0.138	-0.746	0.139
	(0.350)	(0.361)	(0.533)	(0.608)
**Survey Year (2018)**	-0.048	-0.089	-0.571	0.823
	(0.346)	(0.356)	(0.477)	(0.641)
**Survey Year (2019)**	-0.298	-0.430	0.215	-0.765
	(0.429)	(0.447)	(0.715)	(0.731)
**Age**	-0.027^*^	-0.021	-0.026	-0.048
	(0.016)	(0.016)	(0.021)	(0.035)
**Education**	-0.208	-0.260	-1.879^**^	18.603
	(0.596)	(0.607)	(0.876)	(1,890.811)
***Danwei* Support**	1.105^***^	1.187^***^	1.010^**^	1.968^**^
	(0.355)	(0.364)	(0.468)	(0.791)
**Strong Network Strong Social Engagement (SNSE)**		1.00	1.00	1.00
**Strong Network Weak Social Engagement (SNWE)**		-1.894^**^	-2.398^**^	-2.113
		(0.836)	(1.148)	(1.414)
**Weak Network Strong Social Engagement (WNSE)**		13.769	13.400	15.456
		(803.461)	(1,027.803)	(3,956.181)
**Weak Network Weak Social Engagement (WNWE)**		-1.425^*^	-2.227^*^	-1.247
		(0.855)	(1.220)	(1.331)
**Constant**	0.948	2.443^**^	4.954^***^	-15.774
	(0.810)	(1.185)	(1.726)	(1,890.811)
**Observations**	187	187	104	83
**Log Likelihood**	-123.060	-117.551	-60.846	-45.505
**Pseudo R-squared**	0.090	0.161	0.259	0.335

* p<0.1;

** p<0.05;

*** p<0.01.

Model 1 included the control variables. Model 2 analyzed the four types based on the occupational and behavioral characteristics of Changchun’s expatriate high-skilled migrants to reveal the differences in settlement intentions. SNSE types (e.g., university teachers) tended to stay based on their strong social networks and high social engagement.

By contrast, the WNSE type showed lower settlement intentions, with insignificant regression coefficients. This suggests that despite their social engagement, this group lacks sufficient social support owing to weak social networks, which affects their intention to settle. For WNWE types, the results indicated a negative and significant effect, which is consistent with the general lack of local social networks and willingness to engage. In addition, the SNWE-type group (e.g., FAW executives), despite having strong social network support, had lower social engagement and showed non-significant results for settlement intention. Hypotheses 3a and 3c yielded opposite results. For expatriates in the SNWE category, having a strong Chinese social network alone is not sufficient to sustain their settlement intentions. These findings indicate that reduced immigrant social involvement diminishes expatriates’ desire to settle, implying that network strength without engagement may be insufficient to foster long-term residence. Expatriates identified as WNWE who lack strong networks and active social engagement demonstrated markedly lower settlement intentions. This reinforces the concept that insufficient integration within both social dimensions, network and engagement, correlates with a minimal inclination to remain in Changchun.

To compare the differences in willingness to stay in China for high-skilled migrants from countries with different levels of economic development, foreign high-skilled migrants were regressed on the classification by home country, with Model 3 representing migrants from developed countries and Model 4 representing migrants from developing countries. The social participation characteristics of the place of immigration and Chinese network attributes significantly affected the settlement intentions of high skilled migrants from developed countries. Hypothesis 4 verified that there is a country-of-origin difference in willingness to stay; those whose country of origin is a developed country have a lower willingness to stay.

Furthermore, taking high skilled migrants from developed countries with SNSE attributes as reference items, the settlement intention of migrants with WNWE attributes was negatively significant. In addition, SNWE types were associated with a reduction in settlement intention.

### Robustness testing and comparative analysis of differences among high skilled migrants in different cities

In this section, comparison and robustness testing were done (Table 6). To validate the robustness of the findings derived from the Changchun sample, additional analyses were conducted using samples from other cities (Hangzhou, Guangzhou, Yiwu) and a nationwide sample. This approach ensured that the conclusions are not biased by the unique characteristics of Changchun’s social and economic structures.

**Table 6:** Descriptive Statistics of Related Variables, *N*=777, China, 2017–2019.

	Changchun	Hangzhou-Guangzhou-Yiwu	Full Sample
	(Mean/Percent)	(Mean/Percent)	(Mean/Percent)
**Amount**	187	590	777
**Settlement Intention**	1.15	1.48	1.40
**Age**	33.97	35.82	35.37
**Gender**	Male: 72.72% Female: 27.27%	Male: 78.14% Female: 21.86	Male: 76.83% Female: 23.17
**Education**	University -: 6.69% University+: 93.31%	University -: 6.44% University+: 93.56%	University -: 6.69% University^+^: 93.30
**Family Connections**	Yes:74.87% No:25.13%	Yes:74.91% No:25.09%	Yes: 74.90% No: 25.10%
**Individual Social Capital**	Yes: 33.16% No: 66.84%	Yes: 54.07% No: 45.93%	Yes: 49.03% No: 50.97%
**Kinship Support Network**	Yes: 17.11% No: 82.89%	Yes: 18.31% No:81.69%	Yes: 18.02% No:81.98%
**Danwei Support Network**	Yes: 27.27% No: 72.73%	Yes: 11.36% No: 88.64%	Yes: 15.19% No: 84.81%
**Social Participation Behavior**	Yes: 93.05% No: 6.95%	Yes: 88.81% No: 11.19%	Yes: 89.83% No: 10.17%
**Ethnic Interaction Willingness**	3.80	3.84	3.83
**Social Participation Cognition**	Yes: 33.69% No: 66.31%	Yes: 44.07% No: 55.93%	Yes: 41.57% No: 58.42%

To enhance the robustness of the analysis, we subjected the individuals from Guangzhou, Hangzhou, and Yiwu to additional screening and retained only high skilled migrants residing in these cities. This refinement ensured a more precise comparison of the social network and social engagement characteristics of expatriates between northern and southern cities, minimizing the potential bias introduced by the inclusion of individuals not classified as high skilled migrants. By focusing specifically on high-skilled migrants, the analysis offers a clearer understanding of the regional differences in support structures and participation patterns across diverse urban contexts.

Ordinal measurements were used as the dependent variables. The questionnaire item, “How long do you plan to stay in China this time?” was used for measurement, with answer options where “I want to leave as soon as possible” was coded as 1, “I want to stay as long as possible” was coded as 2, and “I want to establish permanent residency” was coded as 3. This approach was used to quantifying the strength of settlement intention [36].

Additionally, we conducted a re-measurement of social networks with a focus on the intentions and behaviors associated with social network construction [37]. First, respondents were asked, “Did you receive assistance upon your arrival in China?” Responses indicating “None/self-arranged travel” were categorized as self-arranged and assigned a value of 1. “Received help from relatives” was labeled as “kinship social network” and assigned a value of 2. Responses indicating assistance from clients, home country’s intermediary company, or Chinese intermediary company were grouped as “unitary social network” with a value of 3. Other responses were assigned a value of 0 to create a multiclass variable. Second, the experience of family members having been to China was identified as the existence of family connections and assigned a value of 1, whereas the absence of such an experience was assigned a value of 2.

Furthermore, we also measured interethnic interaction willingness by asking high skilled migrants whether they were willing to chat with, befriend, marry or allow their children to marry Chinese people. Responses were measured on a five-point scale ranging from “1= *Very willing* to 5=*Absolutely unwilling*,” reflecting a gradient ranging from willingness to engage in basic interaction to deep friendship and even interethnic marriage. These responses were normalized to a range of 1-5 to yield a quantitative variable representing “interethnic interaction willingness.”

Finally, social participation was remeasured, alongside the previously mentioned SoftMax prediction, by introducing a measurement of social participation cognition. We compared “development opportunities,” “family emotional connections,” and “social moral concepts” between China and the home country. Respondents selecting “China is optimal” were marked as 1, while others were marked as 0. The sum of these scores produced a measurement variable for cognition in China. Similarly, respondents selecting “home country is optimal” were marked as 1, while others were marked as 0, generating the measurement variable for cognition of the home country. Ultimately, the SoftMax function was applied for prediction, with the maximum probability indicating the feature of social participation cognition, representing China as 1 and the home country as 0.

The descriptive statistics presented in Table 6 indicate that despite a significant sample sizes disparity between Changchun and the southern cities, the means and percentages of most key variables, such as family ties, kinship support networks, and social participation behaviors, show a high degree of consistency. This consistency suggests that the research sample is highly representative and indicates that high skilled migrants across different cities exhibit similar patterns in terms of social network and behavioral characteristics.

However, two variables- unit support network and personal social capital-show noticeable differences between Changchun and the southern cities. Specifically, the proportion of unit support networks in Changchun was significantly higher than in the southern cities (27.27% vs. 11.36%), while the proportion of personal social capital was relatively higher in southern cities (54.07% vs. 33.16%). These differences may reflect two distinct social support models, with the role of unit support being more prominent in Changchun.

In addition, Yiwu, Hangzhou, and Guangzhou were compared. The results (Table 7) from Model 1 indicate that after controlling for demographic variables, the social support networks of high skilled migrants in Changchun played a crucial role. Particularly, in terms of unit support networks (p<0.01), their own human capital and kinship support networks also exhibited a significant statistical influence, with all coefficients being positive. This suggests that the social support networks of high skilled migrants in Changchun positively impact their living and working environments by providing them with important resources and support. Meanwhile, family connections did not show a significant influence on high skilled migrants in Changchun in the model, indicating that they rely more on their own human capital and the platforms provided by their units, rather than ethnic enclave networks. This finding underscores the importance of individual adaptation to new environments while also highlighting the crucial role of units in providing support and resources.

**Table 7:** Robustness Tests of the Influence of Social Networks and Social Engagement on Settlement Intention, Full Sample, N= 777.

	Settlement Intention		
	Changchun	Hangzhou-Guangzhou-Yiwu	Full Sample
**Age** ^**2**^	-0.0002^*^	0.00004	0.00000
	(0.0001)	(0.0001)	(0.00005)
**Gender**	-0.088	-0.105	-0.112
	(0.175)	(0.098)	(0.086)
**Education**	-0.310	-0.003	-0.046
	(0.297)	(0.166)	(0.145)
**Family Connections**	0.084	0.218^**^	0.191^**^
	(0.183)	(0.093)	(0.084)
**Country Type**	-0.040	-0.110	-0.102
	(0.176)	(0.097)	(0.085)
**Individual Social Capital**	0.352^*^	0.163	0.257^**^
	(0.210)	(0.115)	(0.101)
**Kinship Support Network**	0.122	0.198	0.229^*^
	(0.247)	(0.138)	(0.120)
**Danwei Support Network**	0.741^***^	0.276^*^	0.403^***^
	(0.216)	(0.155)	(0.125)
**Social Participation Behavior**	0.680^**^	0.243^*^	0.323^***^
	(0.304)	(0.131)	(0.120)
**Ethnic Interaction Willingness**	0.015	0.178^***^	0.143^***^
	(0.098)	(0.051)	(0.046)
**Social Participation Cognition**	0.384^**^	0.113	0.180^**^
	(0.165)	(0.083)	(0.074)
**Constant**	1.091^**^	0.509^*^	0.566^**^
	(0.503)	(0.280)	(0.245)
**Observations**	187	590	777
**R** ^**2**^	0.138	0.059	0.064
**Adjusted R** ^**2**^	0.084	0.041	0.050

*Note:*

* p<0.1;

** p<0.05;

*** p<0.01.

Model 3 demonstrated the importance of family connections and their accumulated causal networks. This implies that developed ethnic enclave networks in Southern cities provide high skilled migrants with rich emotional support, which is crucial for their adaptation to and residency in different cultural environments. However, the statistical significance of their unitary support networks was validated, whereas their kinship support networks did not show significance. This indicates that in terms of actual support, the support provided by kinship networks associated with primary social groups is relatively limited. High skilled migrants possesses a relatively high social status and abundant social capital; therefore, they may require more mainstream support and resources, which may extend beyond what ethnic enclave areas can provide. Due to differences in social status and resources, the willingness of high skilled migrants in Southern cities to interact with Chinese people is significant, reflecting their efforts and desires to integrate into the local community and obtain broader social support.

Even transnational immigrants in Changchun were mainly international students. The group seeking transnational career opportunities and career growth was relatively small. Transnational workers in low-end industries are rare, so they cannot form an utterly self-sufficient ecology in terms of food, education, medical care, and job searches like Chinatown in London, Yokohama, and Osaka, Little India in Leicester, and even the African community in Guangzhou; meanwhile, Changchun’s high skilled migrants have substantial human capital, and most work in large organizations such as FAW Group and Jilin University, which provide solid social support in all aspects, including food, clothing, housing, and transportation. We obtained the following information during fieldwork: Regarding accommodation, *danwei* will provide apartments for high skilled migrants. For example, the Changchun Automobile Development Zone launched a construction project for FAW foreign expert apartments with a total area of 194,765 square meters. Moreover, *danwei* always hire translators or establish foreign affairs offices to reduce language barriers for high skilled migrants. They also provide social security and support for spouses and children.

In summary, there are significant differences in the strategies and logic for attracting and integrating high skilled migrants between Changchun and southern cities such as Guangzhou, Hangzhou, and Yiwu. Guangzhou, Hangzhou, and Yiwu. The latter, with their open structures rooted in globalization and diverse communities, are typical representative of China’s economic globalization. These cities attract a large number of high skilled migrants through outward-oriented economies, multinational trade, the internet industry, and export-driven small commodity markets. They also feature relatively mature international communities and ethnic networks, which provide cultural identity, daily life support, and a well-developed “soft” environment for expatriate services. In these southern cities, the social integration of high skilled migrants relies more on ethnic communities and global networks, rather than solely depending on companies or organizations.

Contrastingly, Changchun relies on a stable structure built on the unit system, with traditional economic sectors as the dominant force. The city lacks mature ethnic communities, posing greater challenges for migrants seeking support through cultural similarity or ethnic networks. In Changchun, the core of support lies primarily in the unit support system, which provides high salaries, housing, visas, healthcare, and education for children. High skilled migrants in Changchun are primarily integrated into mainstream Chinese society through the resources provided by their units. Hence, their integration logic is work-centric, rather than being facilitated through ethnic communities or diverse social interactions. Moreover, owing to the relatively underdeveloped living facilities and international service systems in Changchun, expatriates are highly dependent on the services and resources provided by their units for their daily lives and social activities. This dependency further reinforces the dominant role of the unit system.

## Conclusion

Settlement intention reflects immigrants’ thoughts on “remaining” and “returning.” To a large extent, this can predict the next direction of individual mobility and reflect immigrants’ satisfaction with their past migration experiences and outcomes of current urban social interactions. Several extant studies have identified “shallow integration” and “transient household” as common characteristics of foreigners in China. However, as a country that imports immigrants, can China benefit from high skilled migrants despite the emergence of such a psychological state? Moreover, what types of high skilled migrants will choose to stay after arriving in China?

Based on transnationalism and the social network theory, this study first explored the influence of social networks and social engagement in the home and residence countries of high skilled migrants on their willingness to stay.Second, using occupational type and behavioral characteristics, it constructed four ideal types of relative strength of social networks and social engagement, clearly illustrated the differences in the social adjustment patterns and their potential settlement intentions in Changchun and finally expounded the national differences of influencing factors of residence willingness. Building upon this, the study further analyzed the differences between high skilled migrants in the Southern region and those in Changchun, which is located in the old industrial base of Northeast China.

The empirical results indicate that high skilled migrants in Guangzhou, Hangzhou, and Yiwu benefit from well-developed ethnic enclave communities such as the Tianhe’s Guangyue community and the HuiJing community. These communities reduce the migration costs and provide a sense of satisfaction and belonging. However, owing to the limited social support and resources available within these ethnic enclaves, expatriates continue to rely, to some extent, on resources provided by units and organizations. Additionally, they demonstrate a strong desire to interact with mainstream society. Moreover, the presence of foreign-related service systems in these cities significantly enhances the settlement intentions of high skilled migrants.

High skilled migrants in Changchun exhibit uniqueness characteristics. While they possess considerable social support, they lack access to relatively independent and well-developed ethnic enclave communities. Consequently, they rely heavily on the life and social service institutions provided by *danwei* and are embedded in mainstream Chinese society through organizations such as universities, research institutions, and multinational joint ventures. The unit system in Changchun, represented by institutions such as the Foreign Affairs Office and the International Affairs Department, plays a critical role in providing housing, language support, visa assistance, and informational materials to guide expatriate’ stay in China. Furthermore, the *danwei* regularly organize training sessions, cultural festivals, and luncheons that enhance expatriates’ social participation cognition and strengthen their social participation behavior. Significant differences were also observed among expatriates based on occupational types. Migrants with SNSE attributes exhibited stronger settlement intentions, whereas expatriate executives showed relatively lower settlement intentions. The three types of careers in the WNWE category, characterized by smaller social networks and lower social engagement, displayed the weakest settlement intentions.

The contributions are as follows. First, this research extends previous mainstream studies by visiting new empirical groups of North-South mobility and South-South population movements. Second, it enriches the localization-transnationalism framework, and high skilled migrants from different countries of origin are also explored. Finally, the four settlement behavior types reveal the tension between social networks and social engagement among high-skilled expatriates in Changchun. Specifically, social networks provide essential professional and daily life support, and the level of social engagement shapes their motivation for long-term settlement. Overseas professionals are influenced not only by the strength of their social networks but also by the depth of their social engagement. This tension offers a more nuanced perspective on expatriate settlement behavior and provides policymakers with a theoretical basis for developing targeted support measures that facilitate the better integration of various types of high skilled migrants into Chinese society.

Conducting research in northeast China is highly meaningful because it acknowledges the significant socioeconomic differences between the northeast and southern regions. Cities such as Guangzhou, Hangzhou, and Yiwu demonstrate strong international attractiveness and diverse high skilled migrants’ structures. Most high skilled migrants residing in these cities are employed in private institutions. Guangzhou’s high skilled migrants are active in international trade, education, retail, catering, manufacturing, aviation, and services. Contrastingly, Hangzhou’s expatriates are concentrated in the fields of education, trade, and technological innovation, while Yiwu’s expatriates are mainly engaged in international trade. The origin countries of high skilled migrants residing in Guangzhou and Hangzhou are widely distributed across North America, Asia, Europe, Oceania, South America, and Africa, with major migrants exporting countries including the United States, the United Kingdom, Japan, Russia, South Africa, and India. Yiwu has a unique regional appeal that attracts high skilled migrants primarily from Yemen, Pakistan, India, and Middle Eastern countries such as Afghanistan, Iraq, and Jordan. These international high-tech professionals reside in well-developed ethnic communities that help reduce relocation costs and provide a sense of satisfaction and belonging.

Cities such as Guangzhou, Hangzhou, and Yiwu are diverse, and have been the focus of numerous studies, whereas relatively few studies have examined cities with potential, such as Changchun, which has more stable and traditional employment fields and primarily attracts expatriate from Germany, the United States, Canada, Japan, South Korea, and Pakistan. Changchun offers social support but lacks relatively independent and well-developed ethnic enclaves. For instance, the FAW Group provides a comprehensive range of resources, including competitive salaries, high-quality housing, dedicated translators, visa facilitation, and personalized support services, thereby fostering strong organizational ties and professional networks for expatriates. Therefore, migrants are deeply dependent on the life and social services provided by *danwei*, embedding themselves into mainstream Chinese society through universities, research institutions, and multinational joint enterprises.

Research on these issues can provide a better understanding of how *danwei* (work units) support modern society and the relationship between institutional support and high skilled migrants’ mobility. Additionally, local governments and units can play a more proactive role in high skilled migrants attraction and policy formulation by understanding the characteristics and differences of high skilled migrants, and developing scientific and effective high skilled migrants attraction policies to further enhance a city’s technological innovation capabilities and economic competitiveness.

The main findings are as follows: First, the size of social networks has a significant impact on improving the settlement intentions of high skilled migrants in Changchun. Specifically, ethnic and Chinese networks exert a positive impact, whereas Chinese social networks have a negative impact. Second, social participation in places of migration can significantly enhance the willingness of high skilled migrants to stay in Changchun. Migrants who frequently participate in the chambers of commerce and volunteer activities in China, are more likely to become permanent residents. Third, while social networks are often an important starting point for expatriates to integrate into a local communities, their ability to achieve a high level of social engagement determines their willingness to settle. High skilled migrants with strong Chinese networks and social participation attributes are more likely to choose long-term residency. Contrastingly, high skilled migrants with WNWE have significantly lower settlement intentions. Those with reduced social participation characteristics in Changchun also exhibit diminished willingness to stay, and high skilled migrants with SNWE show significantly negative settlement intensions. Fourth, after classifying overall immigration by country of origin, we found that Changchun’s high skilled migrants from developed countries displays unique patterns. Compared to other groups, high skilled migrants with SNSE attributes dominate, whereas those with WNWE or SNWE attributes are significantly less prevalent.

The automobile industry, led by the FAW Group, is an essential pillar of Jilin Province, forming a cluster economy encompassing in all segments of the automobile industry. This necessitate fully exploiting the potential and leading role of high skilled migrants. The social participation rate among high skilled migrants in Changchun was only 16.7% at the time of this study, far below the social participation rate of these expatriates in their home countries, indicating that their potential remains untapped. During our three years of fieldwork, we observed that *danwei* constitute an essential platform for high skilled migrants in Changchun to deepen their social participation in China, thus influencing their willingness to stay. The intermediary role of these work units could serve a critical entry point for transnational group governance and a direction for future research.

However, this study has some limitations. First, we believe that the interlocutors and the content of the interactions in which high skilled migrants engage affect their settlement intentions. Given that China is not traditionally viewed as an immigrant country, the threshold for obtaining permanent residence is relatively high, and the process is complex. High skilled migrants are more likely to formulate the intention to stay in a traditional immigrant country before adapting to its local social behaviors. However, causality cannot be established, as willingness to stay also increases the likelihood of high skilled migrants building social networks and participating in local communities. Furthermore, the data used in this study were obtained from a Changchun dataset and several datasets from southern cities. Therefore, whether the conclusions can be generalized to other cities must to be further verified with data from additional locations.
